# Towards human organ generation using interspecies blastocyst complementation: Challenges and perspectives for therapy

**DOI:** 10.3389/fcell.2023.1070560

**Published:** 2023-01-19

**Authors:** Hemanta Sarmah, Anri Sawada, Youngmin Hwang, Akihiro Miura, Yuko Shimamura, Junichi Tanaka, Kazuhiko Yamada, Munemasa Mori

**Affiliations:** ^1^ Department of Medicine, Columbia Center for Human Development, Columbia University Medical Center, New York, NY, United States; ^2^ Department of Surgery, Johns Hopkins University, Baltimore, MD, United States

**Keywords:** interspecies blastocyst complementation, xenotransplantation, bioengineering, human organ generation, swine model, human pluripotent stem cell, chimera

## Abstract

Millions of people suffer from end-stage refractory diseases. The ideal treatment option for terminally ill patients is organ transplantation. However, donor organs are in absolute shortage, and sadly, most patients die while waiting for a donor organ. To date, no technology has achieved long-term sustainable patient-derived organ generation. In this regard, emerging technologies of chimeric human organ production *via* blastocyst complementation (BC) holds great promise. To take human organ generation *via* BC and transplantation to the next step, we reviewed current emerging organ generation technologies and the associated efficiency of chimera formation in human cells from the standpoint of developmental biology.

## 1 Introduction

Organ transplantation is the ultimate treatment option for patients suffering from refractory diseases. However, there has been an absolute scarcity of donor organs, and the gap between available donors vs. waitlisted recipients continues to expand (Giwa et al., 2017). In 2017, approximately 114,000 patients in the United States waited for an organ transplant (Sykes and Sachs, 2019). Presently, in the United States, another person is added to an organ transplant list every 10 min, 17 people die each day while waiting for donor organs, and approximately 105,800 patients are waitlisted for an organ transplant according to the health resources and services administration (HRSA). To overcome this significant crisis, researchers are investigating various approaches involving direct xenotransplantation, organoids, decellularization, and recellularization, and more recently, organ bioengineering using blastocyst complementation (BC). Depending on the patient’s medical condition, a refractory disease patient also requires an on-time selective option, such as less invasive cellular therapy options or curative organ transplantation that can function immediately after transplantation. In this regard, whole organ generation *via* the BC approach holds great promise with a ready resource (livestock) for cellular therapies and as a radical treatment option for most terminal diseases. However, though BC is emerging as a potential organ transplant option, challenges regarding organ size scalability, immune system incompatibilities, long-term maintenance, potential evolutionary distance, or unveiled mechanisms between donor and host cells remain. These challenges can be overcome by a multifaceted approach, especially by filling in the knowledge gaps on the mechanisms of interspecies chimera formation. In this review, we summarize the history of interspecies chimerism in various animal models to find hints for BC application and describe the challenges and prospects of utilizing BC for human organ generation.

## 2 Hybrids vs. chimeras

In ancient history, humans used the term “chimera” to describe mythical creatures and hybrids. Animals sexually derived from the fusion of gametes from two different organisms, such as mules, are considered “hybrids.” On the other hand, a chimera is defined as an organism in which cells from two or more different organisms have contributed. In nature, hybrids and chimeras are rare due to incompatibilities and developmental divergence during evolution. At the organismal level, evolutionary distance is defined by genome-wide sequence homology between species. Humans, and other species, such as mice, swine, birds, etc., have diverged from one another. A molecular program that causes the interspecific barrier might be independent of genome-wide evolution. Instead, it may depend on the host and donor molecular similarities or distinctions critical for the organogenesis program. For instance, human LIF protein can signal *via* LIF receptors to help maintain a high degree of stemness in mice and rats, but the converse is ineffective (Dahéron et al., 2004). How evolutionary distance affects the formation of hybrids and chimeras is an open question that may vary from situation to situation.

## 3 Interspecies barriers in clone technologies *via* somatic cell nuclear transfer (SCNT)

**FIGURE 1:**
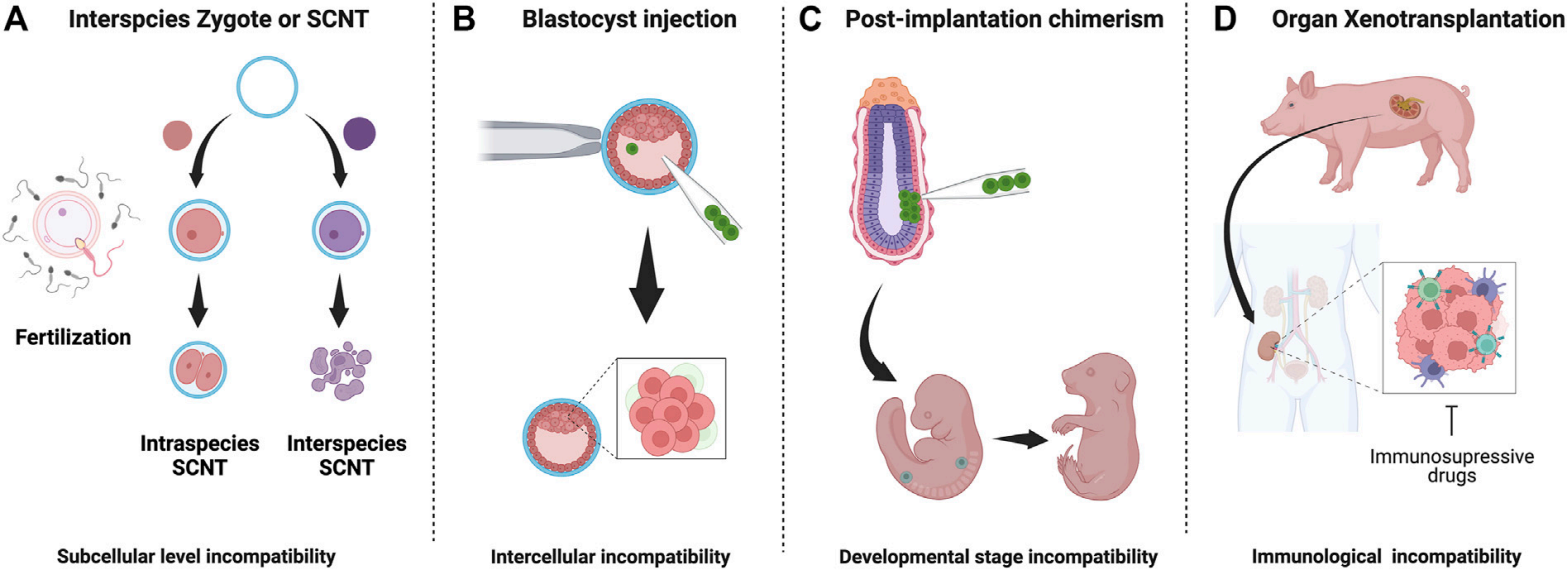
Interspecies embryo incompatibilities and barriers in interspecies chimerism: **(A)** Interspecies zygotes cannot reprogram and fail to develop due to subcellular level incompatibilities **(B)** Cell competition eliminates less fit (donor) cells donor cells in host blastocyst **(C)** Stage-matching of donor cells is challenging and unknown factor contribute to apoptosis of donor cells **(D)** Recipient immune system reacts strongly against graft tissue/organ. Despite long-term immunosuppression, organ failure occurs.

In 1997, a breakthrough discovery was made: Dolly the sheep. Dolly was the first mammal cloned from an adult cell using somatic cell nuclear transfer (SCNT) (Wilmut et al., 1997). This study proved that a differentiated cell has the ability to produce exact copies of its source animals. SCNT has been successfully applied to clone 24 mammalian species, including mice, cows, pigs, cats, rats, dogs, and monkeys (Rodriguez-Osorio et al., 2012). However, SCNT was not successful in interspecies attempts. The process had been tested through electro-fusion using donor somatic cell nucleus replacement into a host’s enucleated interspecies egg cytoplasm, but failed. The failure appears to be due to reprogramming elimination of the donor mitochondria by the host mitochondria, and inability to activate the donor embryogenesis program after fusion (Chang et al., 2003; Chen et al., 2003; St John and Lovell-Badge, 2007; Jiang et al., 2011). The interspecific SNCT studies imply that there are critical interspecies barriers at the subcellular level of totipotent zygotes, distinct from interspecies chimera that are at an intercellular level (Figure 1A). The study of interspecies SCNT, focusing on intracellular compartments, such as the interspecific reprogramming process, mitochondrial competition between donor and host, and genome-stability post-electrical fusion, may open new avenues to better understand subcellular interspecies barriers in SCNT.

## 4 Interspecies barriers in chimeras

The field of chimera research emerged nearly 50 years ago, with pioneering experiments using a mixture of pluripotent stem cells (PSCs) in an intra- or interspecies manner. Briefly, cells from the Ryukyu mouse (*Mus caroli*), when transplanted into blastocysts of the house mouse (*Mus musculus*), showed very high chimerism compared to cells derived from rat blastocysts (Stanny et al., 1980). On the other hand, cells derived from the bank vole (*Clethrionomys glareolus*), a more evolutionary distant rodent from mice, showed no detectable chimerism even during the late embryonic stages (Mystkowska, 1975). Similar studies were also performed within closely related mammalian species such as sheep and goats. Composite blastocysts from goats and sheep could successfully generate goat-sheep chimeras (Fehilly et al., 1984). Likewise, chimeric calves produced from two closely related cow species—*Bos indicus* and *Bos taurus,* were also viable (Williams et al., 1990). These early experiments made a clear distinction between interspecies and intraspecies chimerism. In other words, the evolutionary distance between two species may be inversely correlated with the extent of chimerism (de Los Angeles and Wu, 2022).

This section reviews various interspecies chimera formation rates and chimerism during development. Chimera formation rate refers to the percentage of host animals with donor cells. Assessment of chimera formation requires checking the markers of donor cell survival, proliferation, and differentiation and proof that the donor cells are responsive to the host animal’s developmental program. Chimerism, on the other hand, indicates the % of donor cell contribution to a particular developmental niche or organism. Overall, the efficacy of intraspecies chimerism and chimera formation is far superior to that of interspecies (Table 1). Elucidation of the interspecies barrier incompatibility between host animals and human cells during development is a breakthrough in the field of human organ generation in a host animal.

**TABLE 1:** Chimerism between various donor and host-derived organisms.

#	Donor	Host	Context	Context	% Chimerism	Remarks	Host stage	References	Year	Inter/Intra	Donor cell pluripotency	Donor cell fate in chimera
1	Cattle	Cattle	Germ cells	*in-vivo*	Unknown	WT cattle blastomere derived germ cell in NANOS3-KO cattle	Blastocyst	Ideta et al. (2016)	2016	Intraspecies	naïve	GDF9, VASA, ESR1, NANOS3 positive primordial folicles
2	Chimpanzee	Rhesus macaque	Whole embryo	*ex-vivo*	Unknown	Bcl2 overexpressed chimpanzee ips cell injected into macaque embryo had chimeric contribution	Blastocyst	Roodgar et al. (2022)	2022	Interspecies	primed	Unknown
3	Human	Bovine	Whole animal	*ex-vivo*	8% *	Labelled Human naïve PSCs cultured in NHSM media into bovine blastocysts	Blastocyst	Wu et al. (2017)	2017	Interspecies	naïve/intermediate	SOX2 positive cell
4	Human	Chicken	Neural tube	*in-vivo*	Unknown	Injected hES cells contributed to ectopic nural tube formation in chick embryos	Pre-streak	Martyn et al. (2018)	2018	Interspecies	primed	T(BRACHURY), SOX17 positive cells
5	Human	Monkey	Whole embryo	*ex-vivo*	7% (EPI), 5% (HYP)	extended human ips cell contribute to both embryonic and extra-embryonic lineages in monkey embryos	Blastocyst	Tan et al. (2021)	2020	Interspecies	extended	Epiblast, hyphoblast, extra-embryonic mesenchyme, trophoectoderm lineage
6	Human	Mouse	Whole embryo	*ex-vivo*	<5% *	Region-specified hiPSCS injected into mice epiblast had low chimeric contribution	Epiblast	Wu et al. (2015)	2015	Interspecies	intermediate *	SOX2, FOXA2, T (BRACHURY) positive cells
7	Human	Mouse	Whole embryo	*in-vivo*	<4%	mTOR transient suppresion mediate high chmera of human iPSC in mice	Blastocyst	Hu et al. (2020)	2020	Interspecies	intermediate	AFP, BAND3, RE-COVERIN positive cells
8	Human	Mouse	Whole embryo	*in-vivo*	Unknown	Myd88 knockout human iPSC chimera formation in mice	Blastocyst	Zheng et al. (2021)	2021	Interspecies	primed	CNN1, PAX6, SOX17 positive cells
9	Human	Mouse	Whole embryo	*in-vivo*	Unknown	p53 knockout human iPSC chimera formation in mice	Blastocyst	Zheng et al. (2021)	2021	Interspecies	primed	CNN1, PAX6, SOX17 positive cells
10	Human	Mouse	Whole embryo	*in-vivo*	Unknown	p65 knockout human iPSC chimera formation in mice	Blastocyst	Zheng et al. (2021)	2021	Interspecies	primed	CNN1, PAX6, SOX17 positive cells
11	Human	Mouse	Whole embryo	*ex-vivo*	<5% *	hESCs and hiPSCS injected into mice epiblast had moderate chimeric contribution	Epiblast	Mascetti and Pedersen (2016)	2016	Interspecies	intermediate *	TBX6, SOX1, FOXA2,SOX2, SNAI, TNNT2, PDGFRB, TFAP2A positive cells
12	Human	Swine	Whole animal	*in-vivo*	<1% *	Labelled Human naïve PSCs into swine blastocysts	Blastocyst	Wu et al. (2017)	2017	Interspecies	naïve/intermediate	TUJ1. SMA, CK8, EpCAM, FOXA2 positive ells
13	Human	Swine	Endothelial and Hematopoteic cells	*in-vivo*	<5%	Bcl2 overexpressed hips cell injected into ETV-2-KO swine embryo had chimeric contribution	Blastocyst	Das et al. (2020)	2020	Interspecies	primed	E-CADHERIN, CONNEXIN-43 positive cells
14	Human	Swine	Skelton muscle	*in-vivo*	<5%	TP53-null hips cell injected into MYF5/MYOD/MYF6 -KO swine embryo had chimeric contribution	Blastocyst	Maeng et al. (2021)	2021	Interspecies	primed	MYOD positive cells
15	Human	Swine	Whole animal	*ex-vivo*	6% *	Labelled Human naïve PSCs cultured in NHSM media into swine blastocysts	Blastocyst	Wu et al. (2017)	2017	Interspecies	naïve/intermediate	SOX2 positive cell
16	Monkey	Swine	Whole animal	*in-vivo*	<0.1%	Donor ESCs contributed to various organs of new-born swine-monkey chimera	Blastocyst	Fu et al. (2020)	2022	Interspecies	primed	TBX6, FOXA2, SOX1 positive cells
17	Mouse	Mouse	Immune system	*in-vivo*	100%	Donor mouse cell derived mature B and T cells in Rag2-KO mouse	Blastocyst	Chen et al. (1993)	1993	Intraspecies	naïve	B and T cell
18	Mouse	Mouse	Endothelial and Hematopoteic cells	*in-vivo*	100%	Mouse naïve iPSCs restored PECAM1+ endothelial cells in Flk1-mutant mice	Blastocyst	Hamanaka et al. (2018)	2018	Intraspecies	naïve	PECAM1, a-SMA positive cells
19	Mouse	Mouse	Heart	*in-vivo*	100%	Donor PSCs rescued heart fromation in Nkx2.5-Cre conditional DTA model	8-cell embryo	Coppiello et al. (2022)	2022	Intraspecies	naïve	CD31, CTNL positive cell
20	Mouse	Mouse	Vasculature	*in-vivo*	100%	Donor PSCs rescued heart fromation in Tie2-Cre conditional DTA model	8-cell embryo	Coppiello et al. (2022)	2022	Intraspecies	naïve	LECTIN, CD31 psotive cells
21	Mouse	Mouse	Heart and Vasculature	*in-vivo*	100%	Donor PSCs rescued heart fromation in Nkx2.5-Cre, Tie2-Cre conditional DTA model	8-cell embryo	Coppiello et al. (2022)	2022	Intraspecies	naïve	CTNL, Lectin positive cells
22	Mouse	Mouse	Lung	*in-vivo*	100%	Functional lung generation in Shh-driven Fgfr2 knockout mice	Blastocyst	Mori et al. (2019)	2019	Intraspecies	naïve	NKX2.1, PDPN, SFTPC, SOX2, SCGB1A1, BETA4TUBULIN positive cells
23	Mouse	Rat	Pancreas	*in-vivo*	100%	Mouse donor cell derived pancreas in Pdx1-KO Rat	Blastocyst	Yamaguchi et al. (2017)	2017	Interspecies	naïve	Pancreas
24	Mouse	Swine	Whole animal	*in-vivo*	0%	Labelled Mouse naïve iPSCs had no chimeric contribution in WT swine	Blastocyst	Wu et al. (2017)	2017	Interspecies	naïve	Not applicable
25	Rat	Mouse	Whole animal	*in-vivo*	20%	Labelled Rat naïve ESCs/iPSCs formed chimera in WT mouse	Blastocyst	Wu et al. (2017)	2017	Interspecies	naïve	Brain, heart, intestine, kidney, lung, pancreas, spleen, liver cells
26	Rat	Mouse	Pancreas	*in-vivo*	100%	Rat donor cell derived pancreas in Pdx1-KO mouse	Blastocyst	Kobayashi et al. (2010)	2010	Interspecies	naïve	Pancreas
27	Rat	Mouse	Heart and Vasculature	*in-vivo*	100%	Donor rat PSCs rescued heart fromation in Nkx2.5-Cre, conditional DTA model	8-cell embryo	Coppiello et al. (2022)	2022	Interspecies	naïve	NKX2.5 positive cells
28	Rat	Mouse	Pancreas	*in-vivo*	>20% *	Labelled Rat naïve ESCs/iPSCs formed pancreas in Pdx1-KO mouse	Blastocyst	Wu et al. (2017)	2017	Interspecies	naïve	a-AMYLASE positive cells
29	Rat	Mouse	Heart	*in-vivo*	>20% *	Labelled Rat naïve ESCs/iPSCs formed heart in Nkx2.5-KO mouse	Blastocyst	Wu et al. (2017)	2017	Interspecies	naïve	NKX2.5 positive cells
30	Rat	Mouse	Eye	*in-vivo*	>20% *	Labelled Rat naïve ESCs/iPSCs formed eyes in Pax6-KO mouse	Blastocyst	Wu et al. (2017)	2017	Interspecies	naïve	PAX6 positive cells
31	Rat	Swine	Whole animal	*in-vivo*	0%	Labelled Rat naïve ESCs had no chimeric contribution in WT swine	Blastocyst	Wu et al. (2017)	2017	Interspecies	naïve	Not applicable
32	Swine	Swine	Pancreas	*in-vivo*	100%	WT Swine blastomere derived pancreas in Pdx1-KO swine	Blastocyst	Matsunari et al. (2013)	2013	Intraspecies	naïve	Pancreas
33	Swine	Swine	Endothelial and Hematopoteic cells	*in-vivo*	100%	WT Swine blastomere derived endothelium in ETV2-KO swine	Blastocyst	Das et al. (2020)	2020	Intraspecies	naïve	TIE-2 positive cells
34	Swine	Swine	skelton muscle	*in-vivo*	100%	WT Swine blastomere derived skelton muscle in MYF5/MYOD/MYF6-KO swine	Blastocyst	Maeng et al. (2021)	2021	Intraspecies	naïve	MYF5, MYOD, ACTN2, DES, PAX7 positive cells
35	Swine	Swine	Eye	*in-vivo*	100%	WT Swine blastomere derived eye in MITF-KO swine	Blastocyst	Zhang et al. (2018)	2013	Intraspecies	naïve	PAX6, MITF, BESTROPHIN positive cells

“Host stage” refers to the timing of donor cell injection.

### 4.1 Human chimeras in chick

Chicken models offer significant advantages in the study of developmental biology and chimerism. The developmental stages can be accurately determined, and embryos can be manipulated for experiments such as tissue transplantation, resection, and genetic modification (Hamburger and Hamilton, 1951; Spurlin and Lwigale, 2013; Cloney and Franz-Odendaal, 2015). Early experiments with chick embryos used intraspecific chimeras to test the vertebrate “organizer” concept and its role in gastrulation, known as embryonic differentiation. When transplanted into another, a portion of the anterior streak cells from one embryo gave rise to an ectopic neural plate comprising a neural tube, notochord, and somites (Addington, 1932; Waddington and Schmidt, 1933; Waddington, 1934). Decades later, the first evidence of avian interspecies chimerism was demonstrated by Nicole le Douarin and colleagues by engrafting quail-derived neural crest cells into the neural plate boundary of chick embryos (Couly and le Douarin, 1985). Human embryonic stem cells (hESC) transplanted into the trunk of a chick embryo were also found to differentiate into β-III tubulin-positive neurons in the host microenvironment (Goldstein, 2010). Furthermore, the neurogenic potential of hESCs was tested in mutant chick models of open neural tube defects (ONTDs). ONTDs were significantly rescued in chick embryos by the transplanted hESCs, compared to control groups, as early as post-operative Day3 (Lee et al., 2004). In another study, inoculated donor hESCs adequately contributed to the hindbrain and regions of the spinal cord within host chick embryos (Boulland et al., 2010). Similar results were also observed when hESCs were injected intra-amnionically into neural-tube defective chick embryos after 24 h of lesion induction, miming a clinical situation of human ONTDs (Lee et al., 2006). Recently, in a pioneering effort, human RUES-GLR (germ layer reporter) cells treated with Wnt3a and Activin were transplanted into chick embryos to assess their ability to serve as “organizers”. This transplantation resulted in the generation of an ectopic secondary axis within the chick embryo capable of inducing neural tissue (marked by Sox2/Sox3 expression) in the adjacent regions (Martyn et al., 2018).

These chick studies showed relatively higher chimerism than rodent-human or pig-human chimera, although the exact extent of chimerism was not described. Although the injection timing differed from BC, host chicken embryos were rescued by human PSC-derived cells. They showed differentiation markers with high chimerism beyond the blastocyst stage at the donor cell injection site. Intriguingly, early embryos of humans are discoidal shaped, closely resembling that of chicken, while mouse embryos are more cylindrical (Akhlaghpour et al., 2021). It suggests that the molecular program of early embryos may be similar between humans and chickens, probably related to the polarity and the body plan, allowing human PSCs to differentiate into neuronal lineages. On the other hand, the evolutionary distance between birds and humans is more than 300 million years, while the differences between rodents and humans are about 96 million years old (Nanda et al., 1999; Nei et al., 2001). The implanted chicken niche may have a unique niche character that secretes or expresses proteins or other molecules to overcome interspecific barriers beyond evolution. Suppose these potential mechanisms of human cell compatibility are common in all bird species. In that case, the human chimera studies with giant birds, such as ostrich (*Struthio camelus*), are an intriguing option for studying organ generation. However, birds harbor completely distinct anatomy and circulation systems from humans, which must be considered well. A mouse-avian chimerism examination also would be fascinating to clarify the significance of such physical constraints toward interspecific incompatibility mechanisms in early embryogenesis.

### 4.2 Human chimeras in mice

Chimera efficiency depends on donor cell preparation and host genetic background. Various group attempts have made dramatic progress in maintaining the pluripotency of naïve, primed, intermediated, and extended PSCs under different cell culture conditions. A naïve pluripotent state can be best described by cells within the inner cell mass of a mouse blastocyst. Cells in the morulae or 16-cell stage are considered to have totipotency or expanded potential pluripotency because they can contribute to both embryonic and extra-embryonic lineages. The concept of primed-type pluripotency is typically associated with cells within the post-implantation epiblast of mouse embryos having very low chimeric potential when injected in a host blastocyst. Lastly, the newly established concept of intermediate pluripotency refers to many possible cell states that overlap between naïve-state markers and primed-state markers. In the following section, we discuss how the chimeric potential of PSCs in various pluripotent states varies within host mouse embryos.

#### 4.2.1 Naïve type pluripotency

Regarding donor cell preparation, scientists successfully maintained embryonic stem (ES) cells from mouse blastocysts and recognized their ability to undergo various lineage differentiation over 4 decades ago (Evans and Kaufman, 1981; Martin, 1981; Martello and Smith, 2014). In subsequent years, with refinement in culture conditions, reagents, and specific inhibitors, using a “2i” medium, the combination of a MEK inhibitor and a GK3β inhibitor, mouse PSCs can be maintained and propagated in a state of naïve pluripotency (Ying et al., 2008). For the intraspecies blastocyst injection, a donor cell naïve pluripotent state is fundamentally necessary to achieve a high degree of chimerism (Silva and Smith, 2008). Gene-targeted mouse ES cells have been successfully used in numerous studies for their ability to form germ-line chimeras (Mansour et al., 1990; Bradley, 1991; Robertson, 1991). However, the prolonged culture of PSCs in the 2i medium caused irreversible epigenetic changes leading to defects in developmental potential (Choi et al., 2017; Yagi et al., 2017). To overcome these problems, we have developed a defined media cocktail named a2i VPA LIF, which allows us to maintain PSCs with the highest chimera-forming capacity capable of forming functional lungs (Mori et al., 2019). To generate human naïve PSCs, 5iLAF, ReST, and t2iLGoY mediums have been developed (Theunissen et al., 2014; Pastor et al., 2016; Liu et al., 2017). Further comparative single-cell transcriptome analysis of the induced pluripotent stem cells (iPSCs) cultured in those media showed distinct naïve pluripotency.

#### 4.2.2 Primed type pluripotency

There have been many efforts to generate and maintain various types of PSCs in human subjects. Human ES cells were first characterized as a primed state (Thomson et al., 1998). Unlike mice, human PSCs cannot be sustained in a naïve pluripotent state in the 2i-culture condition (Chan et al., 2013; Gafni et al., 2013; Takashima et al., 2014; Theunissen et al., 2014; Ware et al., 2014; Wu et al., 2015). This fundamental difference is likely a contributing factor behind the low chimerism of human ES in mouse embryos, examples of which are discussed below (Theunissen et al., 2014; Weinberger et al., 2016). The first evidence of human-mouse chimeras came from a study where RUES1 cells were transplanted into mouse blastocysts and examined for chimeric contribution *in-vivo*. Although the extent of chimerism was low, cells of human origin contributed to the prospective foregut epithelium and neuroepithelium at the E8.5 developmental stage (James et al., 2006). In a subsequent study, Hanna and colleagues reported a special 2i-LIF media supplemented with ligands FGF2 and TGFβ1, as well as inhibitors for p38 and JNK, that could confer naïve pluripotency in human ESCs and iPSCs. Additionally, they reported that cells grown in culture conditions robustly contributed to craniofacial tissues and embryonic neural folds within mouse-human chimeras (Gafni et al., 2013). Although this result was acclaimed to be a defining functional read-out for naive human pluripotency, other groups that tested this method failed to generate chimeras (Takashima et al., 2014; Theunissen et al., 2014).

#### 4.2.3 Intermediate type pluripotency

During embryogenesis, cell competition is one of the significant interspecific incompatibility barriers that cause regional elimination of low-fitness cells, accounting for almost 35% of all cells produced *via* the balance between proliferation and apoptosis (Figure 1B) (Clavería and Torres, 2016; Merino et al., 2016; Bowling et al., 2019; Hashimoto and Sasaki, 2020). This mechanism, initially identified in *Drosophila*, involves relatively higher-MYC expressing “winner” cells to dominate the developing embryo by forcing adjacent, relatively lower-MYC “loser” cells to undergo apoptosis (de La Cova et al., 2004; Moreno and Basler, 2004; Clavería et al., 2013; Sancho et al., 2013; Díaz-Díaz et al., 2017). To avoid cell-competition-based elimination of donor cells in a chimeric epiblast, Belmonte and colleagues targeted E7.5 post-implantation mouse embryos for engrafting human PSCs and accessed chimeric efficiency (Figure 1C). By conditioning human ESCs into a region-selective primed state characterized by a transcriptome that closely matches that of a late-streak/no-bud stage mouse embryo, the authors reported efficient chimerism into endoderm, ectoderm, and mesoderm tissues (Wu et al., 2015). Mascetti and Pedersen further illustrated the idea of stage-matching hPSCs with the corresponding mouse embryonic state to enhance chimeric efficiency. However, in contrast to Belmonte’s study, there was no requirement of special pre-treatment for human PSCs before their injection into early and late gastrulation-stage embryos. Yet, primed-type cells could differentiate very efficiently into tissues derived from all three germ layers (Mascetti and Pedersen, 2016). Such disparity in experimental results highlights the extent of technical challenges and the requirement of a reliable genetic or epigenetic landmark for evaluating human PSC lines that form chimera efficiently before injection.

The state of pluripotency can have a profound influence on chimeric efficiency. For instance, primed-type PSCs corresponding to epiblast stem cells cannot form chimeras even in intraspecies contexts (Nichols and Smith, 2009). On the other hand, naïve-type cells show gene expression profiles similar to a blastocyst’s inner cell mass and can contribute to interspecies chimeras (Kobayashi et al., 2010). Although this distinction is well grounded for rodent models, the concept is not well defined in context of large animals (Rashid et al., 2014; Graham, 2017). Wu and colleagues examined all available culture conditions for primed and naïve states of human iPSCs (Wu et al., 2017). In addition to primed and naïve, human iPSCs were also characterized in an intermediate state of pluripotency, previously shown to have chimeric potential in mouse germline (Tsukiyama and Ohinata, 2014). Reporter human iPSCs were amenable to this state of pluripotency and showed significantly higher chimeric contribution in swine embryos compared to cells in other naïve or naïve-like pluripotent conditions (Wu et al., 2017). A recent study also showed that human iPSCs with intermediate pluripotency contribute to primordial germ cell specification in chimeric embryos (Yu et al., 2021).

#### 4.2.4 Extended type pluripotency

In 2017, Deng and colleagues introduced the concept of extended pluripotency, a state of stemness that is transcriptionally distinct from conventional naïve and primed cells. An extended potential state exists between a totipotent 1-cell embryo and a blastocyst in a developing embryo. Both mouse and human extended pluripotent stem cells (EPSCs) were found to have very high chimeric potential, supported by the fact that a single mouse EPS cell could generate both embryonic and extraembryonic lineages *in vivo* (Yang Y. et al., 2017). Further developing this concept, human EPSCs were shown to be approximately 20 times more chimerically efficient than naïve hPSCs when injected into preimplantation mouse blastocysts (Yang Y. et al., 2017). The most recent evidence highlighted the importance of mTOR pathway inhibition in donor human PSCs enabling them to achieve a high degree of chimerism in mouse embryos (Hu et al., 2020; Zhang et al., 2021). In many of the examples discussed above, modified hPSCs were transplanted into a wild-type mouse host, and not surprisingly, the extent of chimerism was low (Freedman, 2018). Based on these results, the human donor cell naïve pluripotency requirement for achieving high chimerism in the host organism is debatable in the human-mouse developmental chimera context. This is because it is difficult to maintain the chimera-competent naïve quality of human iPSCs for the long term even in naïve maintenance medium (5iLAF, ReST, and t2iLGoY) or 2i-medium and the naïve pluripotency is profoundly distinct across the species (Silva and Smith, 2008). Intermediate or extended-type PSCs are attractive; however, substantial efforts are required for efficient chimerism (Yang J. et al., 2017; Gao et al., 2019). How to maintain an organogenic competency in human iPSC, that is, the ability to form functional organs post-injection of cells in the host animals, remains a question, and it may depend on both pluripotency and the host animal developmental program.

### 4.3 Human chimeras in rats

Rats have also been used to study interspecies chimerism in various contexts for developing humanized rat models, although not to the same extent as mice. Early studies in the 1980s utilized the nude rat model to study the engraftment of human skin and immune cells (Vos et al., 1980). These rats exhibited moderate immunodeficiency characterized by loss of lymphocyte counts with age but sustained high counts for neutrophils, eosinophils, and monocytes. Therefore, this model could only support adult human skin grafts for a short time before host-mediated rejection (Brüngger et al., 1984; Gilhar et al., 1986; 1990). Human CD45^+^ cells were detected in various rat tissues, such as the liver, thymus, kidney, spleen, etc., up to 6 months post-in-utero cord blood transplantation (Sun et al., 2007). The success of this model primarily relies on the permissive nature of the immune-deficient fetal state of rats. Recent studies have further perpetuated this concept by showing high engraftment efficiency of human iPSCs and human cancer cells in two rat models of severe combined immunodeficiency (Yang et al., 2018; He et al., 2019; Noto et al., 2020). The degree of immune surveillance for a given tissue also contributes to chimeric efficiency. Regions of the rat brain, such as the corpus striatum, when transplanted by human marrow stromal cells, show a remarkable 20% engraftment efficacy over 72 days with a lack of inflammatory responses (Azizi et al., 1998). In addition to immune aversion, various pathological conditions or tissue injury forms enhance chimerism’s chances. Neural precursors cells derived from human ES cells differentiate into neurons and glial cells in the regions of adult rat brains and spinal cords (Tabar et al., 2005; Yan et al., 2007). Human neural stem cells exert neuroprotective function in rat models of Parkinson’s disease and conditions of stroke-induced ischemia (Yasuhara et al., 2006; Bliss et al., 2007; Andres et al., 2011). Other human stem cell types, such as mesenchymal stem cells, also exhibit localized chimerism in rat organs such as the liver, heart, and pancreas (Grinnemo et al., 2004; Sato et al., 2005; Laporte et al., 2019). These studies primarily attempt to generate humanized rat models of various pathological conditions and tissue damage without using BC methods. With the emergence of new humanized rat models, the future of functional human-rat interspecies chimerism holds excellent promise, while the permissiveness of non-cancerous, normal human epithelial chimerism needs to be addressed well (Agarwal et al., 2020).

### 4.4 Human chimeras in monkeys

Close evolutionary relationships between monkeys and humans make them a suitable model for studying the long-term effects of interspecies organ chimerism (Gibbs et al., 2007; Stevens et al., 2013). Despite their relatively large size compared to small rodents, extended gestation period, and logistical difficulties such as labor and farming costs, monkeys can be valuable models for studying interspecies chimerism in various contexts. Takahashi and colleagues performed human ES stem cell-derived retinal tissue transplantation in two monkey models of injury-induced retinal degeneration (Shirai et al., 2016). It is well known that the odds of immune rejection decrease significantly in human-leucocyte antigen-matched iPSC transplantations (Sugita et al., 2016; Morizane et al., 2017). From this observation, Mandai and colleagues used human iPSCs to generate long-term functioning chimeric retinas in the same primate models of retinal degeneration with lower immunosuppression. In another pre-clinical study, human iPS cell-derived dopaminergic neurons showed functional engraftment in a neurotoxin-induced model of Parkinson’s disease (Kikuchi et al., 2017).

Furthermore, when used in the order of billions, human ES cell-derived cardiomyocytes can regenerate hearts in a non-human primate model of myocardial ischemia (Chong et al., 2014). Despite such success, certain aspects of monkey-human chimerism remain poorly understood. For instance, human ES cell-derived cardiovascular progenitors failed to induce remuscularization in infracted primate hearts (Zhu et al., 2018). From the developmental standpoint, Belmonte and colleagues showed the ability of human EPSCs to differentiate into hypoblast and epiblast lineages in monkey-human chimeras *ex vivo* (Tan et al., 2021). Attempts have also been made to generate monkeys genetically predisposed to immunodeficiency (Niu et al., 2014). Such models can be beneficial in achieving a high chimeric success of engrafted donor cells. However, using monkey-human chimeras for studying complex human brain disorders will require more cautious exploration and robust ethical modalities for crossing the interspecies barrier (Greely and Farahany, 2021). The organ size distinction between humans and monkeys also can be another significant xenobarrier for monkey-human chimeric organ generation, particularly for the solid organs that require anatomically and physiologically functional size for transplantation, such as eyes, bones, kidneys, hearts, lungs, and gastrointestinal tracts.

### 4.5 Human chimeras in pigs

Previously, large animals such as cattle and pigs were mainly used for developing human/animal xenograft models. The main objective was to gain mechanistic insights into graft vs. host tissue interactions that could be leveraged to achieve long-term clinical success of organ transplantation. Modeling such events in smaller animals was not feasible owing to shorter life spans and anatomical disproportionalities. Flake and colleagues showed site-specific differentiation of engrafted human mesenchymal stem cells upon transplantation into sheep embryos (Liechty et al., 2000; Almeida-Porada et al., 2004). Among large animals, pigs have been proposed as an ideal host for studying interspecies chimerism, primarily due to very close anatomical and physiological similarities to humans (Cooper et al., 2016; Nagashima and Matsunari, 2016). The following section summarizes currently available pig models and the prospects of using gene-modified pigs for xenotransplantation (Figure 1D).

## 5 Blastocyst complementation

“Blastocyst complementation (BC)” is a significant concept for organ generation as a regenerative approach. BC utilizes the host developmental program to incorporate donor PSCs. Donor PSCs, when injected into recipient morula or blastocysts lacking critical genes for organogenesis, can replace the defective organ niches and compensate for the functional defects of recipient cells.

Alt and colleagues first illustrated intraspecies BC when they injected wild-type mouse ES cells into RAG2-deficient blastocysts. Mature B and T cells derived from donor PSCs rescued adaptive immune responses in host mice that were niche depleted in terms of lymphocyte diversity (Chen et al., 1993). Approximately two decades later, Nakauchi and colleagues showed the first evidence of interspecies pancreatic organ generation in mice using rat PSCs (Kobayashi et al., 2010). Remarkably, they also showed that the rat pancreatic tissues generated in mice *via* BC were transplantable into a recipient rat disease model and rescued its phenotype (Yamaguchi et al., 2017).

Recently, this concept has been extended to large-animal interspecies, yet other barriers such as donor cell pluripotency, cell-cell competition, and other potential xenogeneic incompatibility barriers result in low chimeric efficacies (Wu et al., 2017; Garry and Garry, 2019; de Los Angeles and Wu, 2022; Roodgar et al., 2022). Chimerism during and beyond the post-implantation stage of development presents a separate set of challenges (Figure 1). Firstly, the stage-matching of donor cells into a particular tissue niche requires extensive optimization to ensure the proliferation of engrafted cells or tissues (Cohen et al., 2018; Thomas et al., 2021). Secondly, donor cells require well-fitness to survive cell competition (Wu and Barbaric, 2021). Finally, tissues derived from donor cells need to avoid immune rejection within transplant recipients even under treatment with immune-suppressive drugs (Lu et al., 2019). Critical barriers in interspecies chimerism corresponding to its developmental stage have been summarized in Figure 1.

### 5.1 Blastocyst complementation in pigs

**FIGURE 2:**
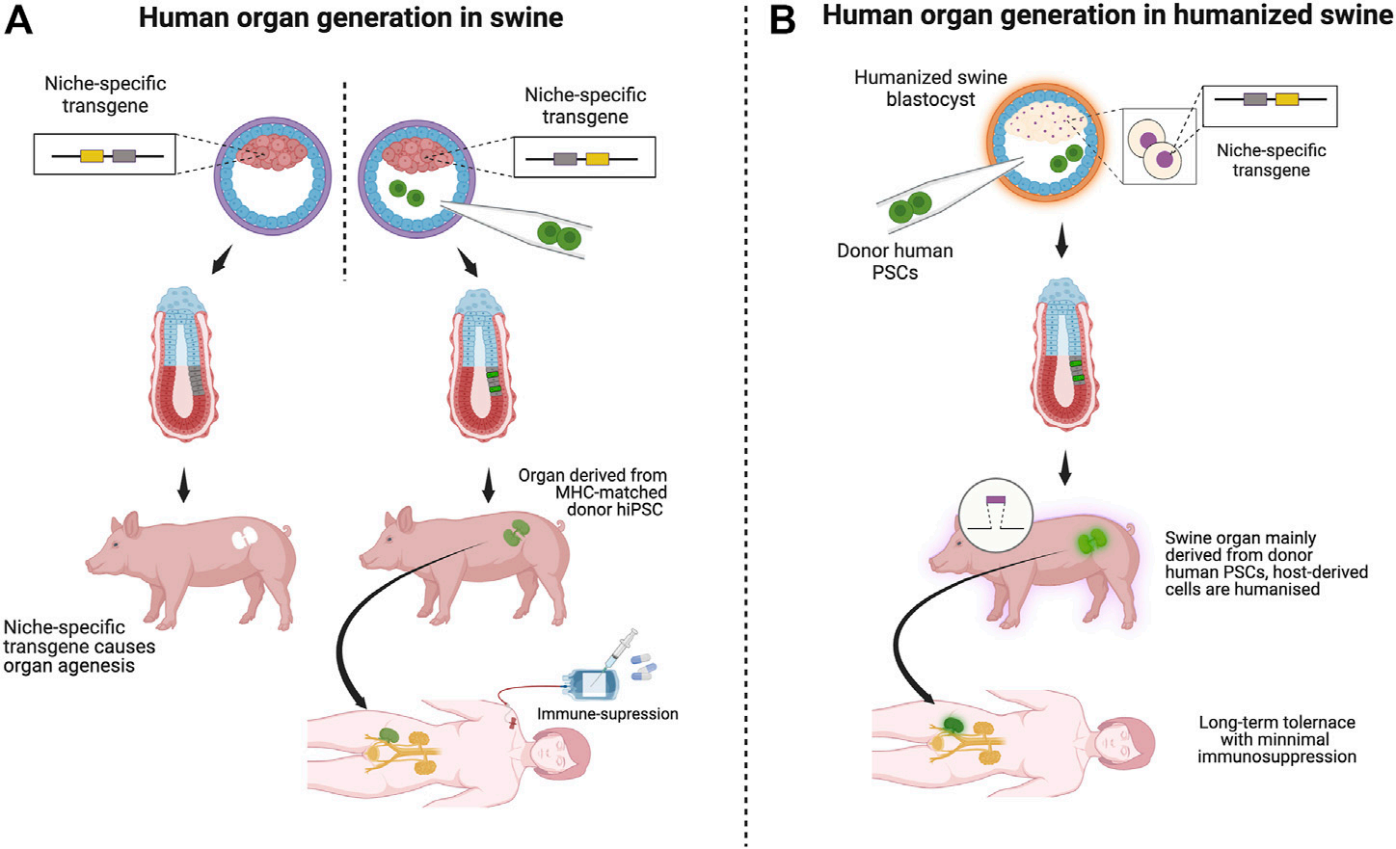
Scenarios of organ transplantation derived from various swine models: Schematic: [**(A)**, left] In BC, vacant organ niche formation by a niche-specific transgene depletion through genome editing is essential. [**(A)**, right] Injection of human patient-derived (or MHC-matched human) iPS cells (green) into a blastocyst of a host animal with an organ failure phenotype. [**(A)**, right, and **(B)**]. **(A)** Transplantation of human organs by BC is unlikely to fail because most cells are of human origin. Immunosuppression may be necessary if the porcine host origin remains in the graft component. **(B)** Organs transplanted from human-derived cells created in humanized pigs are assumed to function long-term in recipients with minimal or no immunosuppression because humanized pigs are designed to minimize immune rejection. Using humanized swine as host animals for the BC will work as a safety guard system for immune rejection.

BC to date has been performed mainly in mice. This is because it is relatively easy to create an organ niche in mice due to the well-established method of creating knockout mice and from the viewpoints of experimental time and cost efficiency. However, the clinical application of BC requires the generation of organs of a size that can be transplanted into humans. BC, with livestock, has attempted to overcome this problem. With newer developments in humanized animal models and an increasingly better understanding of pluripotency across various species, interspecies BC is one of the most promising bioengineering approaches for generating transplantable human organs (Figure 2A) (Garry and Garry, 2021).

Before the advent of CRISPR-Cas9 genome editing technology, pigs had been mass-edited over several decades by classical genome editing methods to accommodate immune systems similar to humans. With the success of CRISPR-Cas9 and its related genome editing technologies, pig-human chimera formation and intraspecies conditional BC approaches, the potential to grow human-size organs in swine is no longer a distant dream (Matsunari et al., 2013; Chang et al., 2018; Hamanaka et al., 2018; Mori et al., 2019; Kitahara A. et al., 2020; Kobayashi et al., 2021; Ruiz-Estevez et al., 2021; Wen et al., 2021). Yet, this will require a three-pronged approach—1) establishment of high chimera competent-donor human ESCs/iPSCs that can survive and propagate within the pig embryo, and 2) generation of a vacant organ niche in swine using CRISPR-Cas9 genome editing. When the pig embryo can genetically be engineered to form a defective organ niche in the targeted organs, cells of human origin can show localized and targeted chimerism to form the respective organ (Rashid et al., 2014; Kano et al., 2022).

Nakauchi and colleagues have successfully created donor-derived pancreas in *PDX1* knockout (KO) apancreatic pigs by BC (Matsunari et al., 2013). This article showed the generation of host pig embryos harboring apancreatic phenotype *via* SCNT, and Kusabira Orange^+^ pig blastomeres complemented the vacant niche. In another study, Nagashima and colleagues generated various organogenesis-disabled phenotypes in organs such as kidneys, liver, and blood vessels by targeting genes like KDR (FLK1), SALL1, and HHEX1, respectively (Matsunari et al., 2020). Recently, Zhou and colleagues successfully complemented the eye using wild-type blastomeres in pig eye deficiency by the mutation of the MITF gene in the host swine (Yao et al., 2021). Garry and colleagues injected human iPSCs into *ETV2* KO pig embryos (Das et al., 2020). Although human cells can incorporate into porcine host blastocysts, many embryos initially rejected human iPSCs. They overcame this issue by overexpressing BCL2, an anti-apoptotic gene, in donor human iPSCs. Remarkably, all TIE2+ endothelial cells were derived from human PSCs in chimeric pig embryos at gestation days 17–18 (Das et al., 2020). This group also reported efficient complementation of TP53 KO human iPSCs in skeletal muscles of MYF5, MYOD, and MYF6 KO pig embryos at two distinct developmental stages—Gestation days 20 and 27 (Maeng et al., 2021). However, the degree of chimerism in these embryos was highly variable, suggesting that interspecies BC needs further optimization and technical consistency.

### 5.2 Blastocyst complementation in large animals

BC methodology in large animals includes swine and cattle. Sendai, Aoyagi, and colleagues generated *NANOS3* KO Wagyu bovines that lack germ cells in ovaries using SCNT. They also succeeded in creating chimeric bovine ovaries capable of producing germ cells derived from donor (*NANOS3* ^+/+^) Holstein blastomeres (Ideta et al., 2016). The advantage of using cattle is the similar gestation period to humans and the potential lower xenogeneic incompatibility *via* interspecific cell competition. Cattle also intrinsically harbor unique tissues identical to humans. For example, limb bones and muscles have better size compatibility with humans than swine. Large internal organ size may also benefit transplantation. However, cattle have lower prolific features for robust applications worldwide than swine, and long-term high-cost performance is the central issue.

In *ex-vivo* culture experimental systems, Belmonte and colleagues reported that human EPSCs cultured in their original medium could contribute to both embryonic and extra-embryonic lineages in ex-vivo-cultured monkey embryos. However, the contribution of human iPSCs is, at best, 7.08% for Epiblast and 4.96% for Hypoblast (Tan et al., 2021). Attempts have been made to form interspecies chimeras among large animals other than humans. Snyder and colleagues recently reported that over-expression of *BCL2* enhances the proliferation of chimpanzees and pig-tailed macaque iPSCs in host rhesus blastocysts. Two days post-injection, most control iPSCs that lacked *BCL2* overexpression did not survive (33.3%, chimpanzee; 54.5%, pig-tailed macaque).

In contrast, almost 100% of *BCL2* overexpressing pig-tailed macaque and chimpanzee iPSCs survived upon injection (Roodgar et al., 2022). These results demonstrate the feasibility of BC as a method to create human organs in large animal organ niches or non-human primates. In contrast, the efficiency of chimera formation with the versatility of genetic modifications in large animals remains low compared to attempts in rodents. Most current studies limit our understanding of chimerism to early developmental stages. Hence, it would be interesting to study interspecies chimerism over various developmental stages—from blastocysts to post-implantation- to validate the effectiveness of host models and donor cells.

Although very promising, successful BC doesn’t guarantee functional organ generation. Until recently, only three organs—the brain, the lung, and the pancreas, have been successfully complemented in a way that has produced viable adult animals phenotypically indistinguishable from that of a WT littermate (Kobayashi et al., 2010; Chang et al., 2018; Mori et al., 2019). The barrier of functional organ generation is even higher in the case of interspecies chimeras. The only functional organs generated *via* interspecies chimerism are the pancreas and thymus (Kobayashi et al., 2010; Isotani et al., 2011). Generation of functional organs by intra- or interspecies BC requires not only compatible donor cells but also appropriate host models in which the function of each target organ can be assessed in live adult animals (Kobayashi et al., 2010; Chang et al., 2018; Mori et al., 2019).

## 6 Swine as a model for xenotransplantation

In 1964, Dr. James D. Hardy performed one of the first xenotransplantation surgeries in modern medicine by replacing a 68-year-old patient’s heart with that of a chimpanzee. Although that first patient did not survive beyond 2 h and succumbed to a hyperacute immune response, there has been tremendous progress in the field of xenotransplantation over the last sixty years: earlier this year, the first porcine-to-human heart transplantation was performed in a patient who was not a candidate for conventional allogeneic heart transplantation, and the patient survived for 2 months (Griffith et al., 2022). Much progress may be attributed to a deeper understanding of the immune system and the relative ease of gene editing (Ekser et al., 2017; Niu et al., 2017). Although many large animals have been considered, pigs are more favored for xenotransplantation mainly because of ease of breeding (with large and frequent litters) and physiologic organ and size similarity to humans. The grafts of porcine origin survived for months and, in some cases, even years upon transplantation into non-human primates. However, xeno-organ transplantation to monkeys, such as lungs, islets, heart, and kidneys, requires systemic immune suppressive drugs for the long term, and most organs are ultimately rejected (Sykes and Sachs, 2019). Strikingly, the thymokidney approach can induce tolerance across allogeneic barriers, prolong the life of the xenograft when transplanted across xenogeneic barriers, and may ultimately be used to reduce the intensity of immunosuppression for xenotransplanted organs (Yamada et al., 1999; 2000; Shimizu et al., 2005; Rivard et al., 2018).

If an organ from a wild-type pig is transplanted directly into a human patient or a non-human primate, the organ will be rejected within minutes. This form of rejection, also known as a hyperacute immune reaction, results from interactions between host-preformed natural antibodies (Nabs) with the galactose-α1,3-galactose (Gal) antigens expressed on cells of pig grafts (Platt et al., 1991). Using antibody absorption techniques, acute rejection can be delayed to a few hours, but eventually, the graft is rejected (Kozlowski et al., 1997). To overcome innate responses and non-Gal preformed antibodies in addition to T Cell dependent responses against graft, researchers have continued to modify the pig genome to 1) target non-Gal natural antibodies and 2) correct species incompatibilities between swine and primate coagulation, phagocytosis and complement regulatory proteins. These include coagulation inhibition by overexpression of anti-coagulation factors hCD39 and hCD141, that express on the surface of endothelial cells, as well as the addition of complement regulatory protein human transgenes (Lee et al., 2018; Singh et al., 2019; Kemter et al., 2020). Using serial SCNT, additional mutations have been accommodated in pigs to avoid anti-inflammatory and anti-apoptotic responses (Fischer et al., 2016). For details on various engineering strategies of an ideal humanized pig, please refer to the latest reviews by Qin and colleagues (Lu et al., 2020).

If permissive, the humanized swine model would be an ideal background for generating niche-depleted host models using the BC approach (Figure 2B). Upon complementation by donor human PSCs, the resulting organ would be suitable for humanized organs, the mixture of human cells and humanized host cells, into MHC-matched patients awaiting organ transplantation. Suppose the entire targeted organ niche that involves endoderm, mesoderm, and ectoderm derivatives would be complemented by human iPSC-derived cells. It is an ideal treatment for MHC-compatible patients awaiting organ transplantation, with minimal or no immunosuppressive approaches.

Transplanted organs should theoretically be superior to humanized porcine-derived organs in function and means of evading short- and long-term immune responses since all cells are derived from each individual patient or MHC-matched donor (Figure 2B).

## 7 Other efforts for organ generation

With the introduction of iPSC cell technology, the field of organoid biology expanded to provide simple yet powerful insights underlying human tissue development and, more importantly, disease modeling of pathophysiological conditions (Rossi et al., 2018; Kim et al., 2020). Various organs, such as the liver, lung, kidney, brain, intestine, and even specialized tissues such as the endometrium, have been modeled using human iPSC-derived organoids, histologically similar to human organs (Lancaster et al., 2013; Takasato et al., 2015; Turco et al., 2017; Fujii et al., 2018; Hu et al., 2018; Sachs et al., 2019). With the increasing success of human iPSC-derived organoid grafting studies, exploring organoid engraftment in a non-human host such as a mouse is exciting. Liver and kidney organoids derived from human iPSCs induced vascularization from host mouse tissue and support host tissue function (Takebe et al., 2013; van den Berg et al., 2018). Likewise, human intestinal organoids showed robust viability when implanted into mouse mesenteric tissues (Cortez et al., 2018). In another set of studies, human iPSC and ESC cell-derived brain organoids established subcortical projections and functionally integrated them into the mouse pre-existing neural (Dong et al., 2020). Human neural crest cells differentiate into melanocytes and produce pigmented hair in a c-kit mutant mouse background that lacks melanoblasts (Cohen et al., 2016). Engraftment of human iPSCs-derived neurospheres recover motor function characterized by synapse formation and increased local myelination in a nude mice model of spinal cord injury (Nori et al., 2011). In two models of acute liver failure, human liver organoids derived from hiPSCs rescued hepatic functions and improved survival rates in mice (Nagamoto et al., 2016; Nie et al., 2018). Human ES cell-derived cerebral organoids, when transplanted into injured mouse cerebral cortices, showed high survival efficiency, robust vascularization from host tissues, and recovery of axonal projections along corticospinal tracts (Daviaud et al., 2018; Kitahara T. et al., 2020). Transplantation of human iPSC-derived endothelial-like cells in a mouse model of hindlimb ischemia caused significant improvement in postnatal vascularization (Cho et al., 2007). The engineered human iPSC-derived organoid transplantation into the defective tissue niche, particularly in the immune-privilege niche such as subrenal regions, testes, eyes, liver, and brain, holds great promise for evaluating stemness and mouse disease modeling as a next-generation personalized medicine (Harrison et al., 2014; Paquet et al., 2016; Driehuis and Clevers, 2017; Artegiani et al., 2020). Overall, human iPSC-derived organoids are compelling disease models *in vitro* and *in vivo*. However, the main challenges of organoid.

-based approaches are the lack of transplantation methods, scalability to generate whole organs, and reproducibility to generate all cell types to replicate organ function. Unless these issues are resolved, the feasibility of organoids for organ transplantation is low and will depend largely on the stage of the disease; the earlier, the better, but not likely, at the terminal stage.

Tissue engineering is another approach to generating transplantable organs. In such an approach, the goal is to grow an entire or partial organ *in vitro*. If an engineered organ could be derived from autologous cells of the recipient, the chances of rejection would be close to zero. Creating mechanically, anatomically, physiologically, and biologically compatible organs similar to native organs is ideal. In this regard, decellularization of the extracellular matrix preserves the vascular network and structural framework (Crapo and Wang, 2011). In theory, a decellularized organ scaffold would function as a barcode of cellular repopulation. After an optimal number of cells have populated an organ scaffold, a bioreactor is used to facilitate organ generation. Bioreactors are devices where biological and/or biochemical processes develop under closely monitored and tightly controlled environmental conditions (Martin et al., 2004). Decellularized scaffolds have been made for various organs, including the lung, heart, kidney, liver, intestine, bladder, corneas, limbs, pancreas, and vasculature. (Ott et al., 2008; Hashimoto et al., 2010; Petersen et al., 2010; Yang et al., 2010; Quint et al., 2011; Totonelli et al., 2012; Song et al., 2013a; Gerli et al., 2018; Goh et al., 2019).

For lung tissue engineering, Niklason and colleagues repopulated acellular rat lung scaffolds with neonatal rat lung epithelial cells and microvascular endothelium. They generated viable lung tissue for up to 1 week under *in-vitro* conditions (Petersen et al., 2010). Ott and colleagues reported that perfusion decellularization of cadaveric lungs yield intact scaffolds seeded with cells to generate bioartificial lung grafts. Upon transplantation of such bioartificial lungs into rats, the grafts maintained respiratory function for up to 7 days (Song et al., 2011). Large animal trials have also been conducted with the hope of pre-clinical applications. Cortiella and colleagues generated porcine lung scaffolds with autologous cells from porcine recipients. Within 2 weeks of transplantation, the lung grafts generated alveolar tissues with supporting vasculature without any sign of immune rejection (Nichols et al., 2018). Ott and colleagues seeded porcine decellularized lung scaffolds with human airway epithelial progenitor cells derived from human donor lungs and banked human umbilical vein endothelial cells. By repopulating porcine extracellular matrix scaffolds with human endothelial cells, they generated pulmonary vasculature with mature endothelial lining supplemented with anti-thrombotic function to enable blood perfusion. They created a functioning gas exchange graft by repopulating the epithelial surface with human epithelial progenitor cells. This graft could withstand physiological blood flow from the recipient’s pulmonary circulation and exchanged gases upon ventilation during the 1-h after transplantation (Zhou et al., 2018).

Initial studies on kidney tissue engineering focused on tissue decellularization methods. Soker and colleagues decellularized porcine kidney scaffolds using a technique that sustained blood pressure up to 2 weeks post-implantation into recipient swine (Orlando et al., 2012). Decellularized monkey kidneys were also functionally effective and biocompatible (Nakayama et al., 2010). Like the lung, kidney tissue engineering also requires the recellularization of decellularized scaffolds, as shown by various studies using swine, monkey, rat, and human organs. Upon transplantation, bioengineered kidney perfused well after orthotopic transplantation into anephric rats and showed urine production (Song et al., 2013b).

Taylor and colleagues reported perfusion decellularization of the whole rat heart, thus setting the stage for other groups to examine this for large animals such as swine (Ott et al., 2008; Wainwright et al., 2010; Weymann et al., 2011; Momtahan 2016). Human-sized decellularized porcine hearts were also developed and showed recellularization of coronary vasculature and myocardium with measurable electrical activity in the decellularized scaffold (Weymann et al., 2014). Shimizu and colleagues reported heterotopic transplantation of a decellularized porcine heart scaffold with mesenchymal stem cells into recipient pigs. The scaffolds could endure surgical procedures and perform short-term coronary artery perfusion by angiography (Kitahara et al., 2016).

Bioengineering techniques of organ generation involving decellularization-recellularization approaches can be challenging in many ways. For instance, existing protocols cannot guarantee the complete removal of residual toxic products within a decellularized tissue. Furthermore, upon transplanting bioengineered tissue into a recipient organism, the degradation rate of decellularized scaffolds should be synchronous with connective tissue remodeling to prevent transplantation failure. Yet, despite its limitations, bioengineering holds excellent promise for organ generation. Further efforts for optimizing recellularization coupled with drug-or genome-editing-mediated approaches for long-term organ maintenance, particularly endothelial components, are essential for organ transplantation into patients suffering from refractory diseases.

## 8 Future perspectives

Various chimeric, hybrid, and bioengineering studies are being attempted in many initiatives that could be applied to the generation of human organs. Still, most fields are immature, but the successful impact is enormous. Among them, BC is one of the most promising technologies, especially for generating whole organs for transplantation. One of the critical elements for successful entire organ generation using BC is a better understanding of the mechanism of interspecies chimerism between human and host cells during the host developmental program. However, the studies of human-animal chimeras are limited by a lack of resources and ethical concerns. In particular, the contribution of human PSCs to neurogenesis and reproductive tissues within chimeric animals can have profound ethical implications (Bourret et al., 2016; Kwisda et al., 2020). These concerns can be mitigated by engineering cells to be incompetent of ethical-related lineage differentiation.

Another essential step for BC’s success is preparing human iPS cells with organogenic potential. Comparative genome-wide and epigenome-wide analysis of host animal pluripotency with human iPS cells can provide critical guidance. Besides human pluripotency maintenance in human iPSCs, ES cells have also recently been derived from large animals such as pigs, cows, and sheep (Bogliotti et al., 2018; Choi et al., 2020; Vilarino et al., 2020; Kumar et al., 2021). However, except for one study involving pig blastomeres, it has been technically challenging to accurately define naïve state pluripotency and germline competence of large-animal stem cells (Matsunari et al., 2013). To overcome the incompatibilities at the molecular level for efficient interspecies chimerism and subsequent xenotransplantation, humanized large-animal models need to be established, and attempts are already beginning to emerge (Yang et al., 2016; Boettcher et al., 2020). Most recently, Wu and colleagues showed further evidence for the involvement of interspecific cell competition during early embryogenesis. This paper demonstrated the inflammatory-like pathway activation of Myd88-p65-NFkB signaling in donor human iPS cells. The loss of function of Myd88 resulted in overcoming cell competition in early embryogenesis (Zheng et al., 2021). The common feature of outcompeting donor intra- and interspecies PSCs, including ES and iPSC cells that form functional organs in host large animals, is still an open question.

Bioengineered organ transplantation is another option to solve the shortage of donors. Toward human transplantation, the generation of scaffolds based on swine adapted to human organ sizes or human iPSC-derived organoids is promising. However, the fabrication of whole organs that can withstand clinical application has not been realized. Further conceptual and technological breakthroughs are needed beyond the scaffold barcodes of decellularized organs and the self-assembling function of organoids. In this regard, the BC method has perspective advantages over the organoid and decell-recell approaches in terms of scalability, livestock, and a surgical indication of organ transplantation applicable to most end-stage refractory diseases. The advantage of decell-recell-based or organoid systems over the BC approach is the potential for a xeno-free environment and less invasiveness during transplantation. In the future, depending on the recipient’s refractory disease status, the advantages and disadvantages of each technology should complement each other to provide us with multiple beneficial options.
